# Chloride channel accessory 4 suppresses stem cell-like properties of colorectal cancer and enhances anti-PD-1 immunotherapy

**DOI:** 10.1016/j.gendis.2025.101859

**Published:** 2025-09-20

**Authors:** Fang Wei, Qi Zou, Qihui Sun, Tingting Jiang, Tian Cai, Xiaojia Li, Keping Xie

**Affiliations:** a Center for Pancreatic Cancer Research, The South China University of Technology School of Medicine, Guangzhou, Guangdong 510006, China; b Department of Immunology and Pathology, The South China University of Technology School of Medicine, Guangzhou, Guangdong 510006, China; c Guangzhou First People's Hospital and The Second Affiliated Hospital, South China University of Technology School of Medicine, Guangzhou, Guangdong 510180, China

**Keywords:** Cancer stem cell, CLCA4, Colorectal cancer, Immunotherapy, Vimentin

## Abstract

Reduced chloride channel accessory 4 (CLCA4) levels are linked to cancer development, while its role and mechanism in cancer stem cells (CSCs) remain unclear. In this study, we discovered that decreased CLCA4 expression was evident in CD133^+^CD44^+^ colorectal CSCs and chemoresistant colorectal cancer (CRC) cells. Increased expression of CLCA4 inhibited the expression of stemness genes, reduced tumorsphere formation, suppressed the self-renewal, migratory, and invasive capabilities of colorectal CSCs *in vitro*, and suppressed the tumorigenicity of colorectal CSCs *in vivo*. Mechanistically, CLCA4 interacted with vimentin, leading to FAK pathway inactivation and subsequent suppression of CSC expansion, while vimentin up-regulation attenuated the effects of CLCA4 down-regulation and established its role in CLCA4-mediated colorectal CSC self-renewal. Decreased CLCA4 expression was positively correlated with colorectal CSC markers and vimentin in clinical specimens. Increased CLCA4 expression promoted the infiltration of cytotoxic CD8^+^ T cells and enhanced the anti-PD-1 therapeutic efficacy. Our findings suggest that CLCA4 could impede colorectal CSC self-renewal by interacting with vimentin to suppress the FAK signaling pathway, potentially reducing tumor cell stemness and evading immune surveillance. The new findings on cellular and molecular mechanisms underpinning CRC development and progression could offer new perspectives for potential intervention and treatment of CRC.

## Introduction

Colorectal cancer (CRC) is one of the most common cancers worldwide, accounting for over 1.8 million new cases and 880,000 deaths annually.^1^ In China, the incidence and mortality of CRC have been rising rapidly in recent decades, posing a major public health concern.^2^ Many genetic, epigenetic, and environmental factors contribute to CRC pathogenesis. Although diagnostic and therapeutic modalities for CRC have advanced considerably, prognosis remains dismal, particularly for those diagnosed at late clinical stages.^3^ Tumor recurrence and metastasis after surgery are major obstacles to therapeutic response and long-term survival in CRC.

A subpopulation of cancer cells with stem cell-like features, termed cancer stem cells (CSCs), has been identified in CRC and implicated in treatment resistance, metastasis, and relapse.^4^ CSCs possess unlimited self-renewal and differentiation capacities and increased ability to invade normal tissues and to evade immune surveillance.^5^ Therefore, elucidating molecular determinants and signaling pathways governing CRC stemness is crucial for developing effective targeted therapies.

The calcium-activated chloride channel regulator (CLCA) family represents a class of self-cleaving, secreted proteins that modulate calcium-dependent chloride currents.^6^ Aberrant expression of certain CLCA members has been observed in multiple cancer types, suggesting that they may play a role in tumorigenesis. For instance, CLCA2 acts as a tumor suppressor in colon cancer and breast cancer.^7^^,^^8^ CLCA4, as a calcium-activated chloride channel regulating protein, is associated with the development of various cancers, including CRC and hepatocellular carcinoma.^8^^,^^9^ Down-regulation or mutation of CLCA4 may affect tumor progression. Nevertheless, the mechanistic role of CLCA4 in colorectal CSCs remains unknown.

In recent years, the introduction of immune checkpoint blockade antibodies has represented a significant therapeutic advancement for a spectrum of malignancies. Antibodies targeting programmed cell death protein 1 (PD-1), along with cytotoxic T-lymphocyte-associated antigen 4 (CTLA-4), have demonstrated significant clinical benefits in certain patient populations, bringing new optimism to CRC treatment.^10^, ^11^, ^12^ Despite these advances, evidence from recent preclinical studies suggests that the efficacy of immune checkpoint blockade monotherapy in CRC is not as robust as desired.^13^ This has prompted an exploration into the integration of immune checkpoint blockade with other treatment modalities within combinatorial therapeutic strategies, with the hope of achieving synergistic effects. Moreover, there is a growing body of evidence indicating that immunotherapy may lead to an enrichment of tumor cells with CSC properties, potentially contributing to adaptive resistance and disease relapse.^14^ The role of CLCA4, a protein of interest in CRC, in mediating stemness and its potential to enhance the efficacy of immunotherapy in CRC patients, remains an open question.

Here, we investigated the synergistic potential of CLCA4 overexpression in combination with immune checkpoint blockade to induce remission in CRC and to explore the mechanisms by which CLCA4 may influence stemness transformation during CRC progression.

## Materials and methods

### Colorectal cell lines

SW480 and SW620 human CRC cell lines and CT-26 murine colon cancer cells were cultured in RPMI 1640 growth medium supplemented with 10% fetal bovine serum. The cells were incubated at 37 °C in a humidified 5% CO_2_ atmosphere to maintain optimal growth conditions. All the cell lines were authenticated using short tandem repeat profiling analysis, routinely tested for mycoplasma contamination within the last 6 months using Hoechst staining and PCR, and used at passage numbers <10 for this study after reception or thawing in our laboratory.

### Generation of 5-fluorouracil and cisplatin-resistant SW480 cells

To develop 5-fluorouracil (5-FU) and cisplatin-resistant SW480 cells, increasing drug concentrations were incrementally applied. Briefly, SW480 cells were seeded in 60-mm plates for 24 h before adding 10 μM 5-FU or 10 μg/mL cisplatin for 48 h. The IC50 values were found to be approximately 20 μM for 5-FU and 20 μg/mL for cisplatin. To ensure that the cells were exposed to sub-lethal but effective concentrations, 10 μM for 5-FU and 10 μg/mL for cisplatin were chosen as the starting concentrations for the drug resistance induction. After replacing the medium lacking the drug and allowing cells to reach 90% confluence, the dose was doubled. This incremental increase in 5-FU (10–80 μM) or cisplatin (10–80 μg/mL) selection occurred over 2 months. Surviving cells exhibiting resistance to 80 μM 5-FU or 80 μg/mL cisplatin were harvested and termed drug-resistant and then used in subsequent experiments.

### Clinical samples

After obtaining written informed consent and ethics approval from Guangzhou First People's Hospital, primary CRC specimens and matched adjacent normal tissues were collected. For quantitative real-time PCR analysis, 20 CRC and 20 adjacent normal samples were obtained. Additionally, 100 CRC samples were collected to analyze the correlation between CLCA4 expression and stem cell marker levels by immunohistochemistry. The uses of specimens in this study were authorized by the Guangzhou First People's Hospital Ethical Review Committee (No. K-2019-009-01).

### Tumorsphere formation assay

The CRC cells were harvested, enumerated, and subsequently plated at a density of 5000 cells per well in six-well ultralow attachment plates (Corning). The culture medium used was serum-free DMEM-F12 (Gibco) supplemented with epidermal growth factor (20 ng/mL, PeproTech), basic fibroblast growth factor (10 ng/mL, PeproTech), and B27 (1:50 dilution, BD Biosciences). Following a week-long incubation, the formed tumorspheres were quantified using a phase-contrast microscope.

### Transwell migration and Boyden invasion assays

For the transwell migration assay, 1 × 10^5^ cells were plated in the upper chamber (8.0 μm pores, BD Biosciences) using serum-free RPMI 1640. For the Boyden invasion assay, Matrigel (BD Biosciences) was applied to the upper chamber. The lower compartment contained RPMI 1640 with 10% fetal bovine serum as the chemoattractant. Following 48-h incubation, cells that migrated or invaded were fixed with 100% methanol, stained with hematoxylin (Sigma), and quantified in five random fields.

### Quantitative real-time PCR

Total RNA was extracted from CRC cells using TRIzol reagent (TaKaRa). Subsequently, cDNA synthesis was performed using the PrimeScript RT reagent kit (TaKaRa) according to the manufacturer's recommended protocol. Quantitative real-time PCR analysis was conducted using the SYBR Green Premix ExTaq kit (TaKaRa). The specific primer sequences used in PCR are listed in Table S5. The expression levels of target mRNAs were normalized to GAPDH, a commonly used reference gene. The fold changes in mRNA expression were determined using the 2^–ΔΔCt^ method.

### Western blotting analysis and antibodies

The cells were harvested and lysed using RIPA buffer supplemented with protease inhibitors and phosphatase inhibitors. The resulting protein lysates were separated by SDS-PAGE and transferred onto a PVDF membrane (Millipore). The membranes were sequentially incubated with primary antibodies specific to the targets of interest, as listed in Table S6, followed by incubation with horseradish peroxidase-labeled secondary antibodies. The detection of protein bands was achieved using enhanced chemiluminescence (CWBIO Technology). To ensure equal protein loading, GAPDH was used as the internal control.

### Mass spectrometry analysis

Tryptic peptides were analyzed using a nanoUPLC-ESI-LTQ Orbitrap mass spectrometer (Thermo Fisher Scientific) following the manufacturer's recommended protocol. Only proteins identified by at least two unique peptides were considered. Proteins identified by the same set of peptides were grouped together. The relative abundance of each protein was estimated using the normalized spectral abundance factor method.

### Immunoprecipitation analysis

Immunoprecipitation was performed to isolate protein complexes from cell lysates using purified antibodies on an agarose support. Briefly, cells were lysed in ice-cold immunoprecipitation lysis buffer (50 mM Tris–HCl, pH 7.4, 150 mM NaCl, 1% NP-40, 0.5% sodium deoxycholate, 0.1% SDS, and protease inhibitors). The lysates were centrifuged at 12000 *g* at 4 °C for 10 min, and the supernatants were collected. The supernatants were incubated with the appropriate antibodies at 4 °C for 2 h with gentle rotation. Protein A/G agarose beads were then added to the lysates and incubated at 4 °C for an additional 1 h. The beads were washed three times with ice-cold immunoprecipitation lysis buffer and then resuspended in the sample buffer. The samples were boiled for 10 min and then subjected to Western blotting analysis.

### GST pull-down assay

In the glutathione-S-transferase (GST) pull-down assay, His-Vimentin and GST-CLCA4 fusion proteins were expressed in *E. coli* and purified. The purified GST-CLCA4 or GST control was combined with His-Vimentin and incubated overnight at 4 °C, and then washed to remove any unbound protein. The proteins were then separated by 10% SDS-PAGE and analyzed by Western blotting to detect His-Vimentin and GST-CLCA4. The interaction was confirmed by the presence of His-Vimentin in the GST-CLCA4 lane on a Western blotting, indicating that His-Vimentin binds to GST-CLCA4.

### Lentivirus production and transduction

The *pLV-CLCA4-Flag-puro* plasmids and *pLV-vimentin-HA-puro* plasmids were purchased from GeneCopoeia, and the lentiviral packaging plasmids *pMD2.G* and *psPAX2* were provided by Prof. Keping Xie (The South China University of Technology School of Medicine, China). To generate lentiviruses, this lentiviral vector plasmid was then co-transfected into 293T cells along with packaging plasmids. The transfected 293T cells then expressed the viral proteins, assembled non-replicating lentiviral particles, and released them into the supernatant. This lentivirus-containing medium can be collected and purified as a high-titer stock for transduction of target cells.

To generate stable CRC cell lines, SW480 and SW620 cells were transduced with the lentiviruses and incubated for 72 h. Subsequently, the transduced cells were selected with 5 μg/mL puromycin. To validate the transduction efficiency of the lentiviruses, the mRNA and protein expression levels were examined by quantitative real-time PCR and Western blotting.

### Mouse xenografts for tumor experiments

The animal study was approved by the Institutional Animal Care and Use Committee at South China University of Technology. Athymic nude mice obtained from the Medical Laboratory Animal Center of Guangdong Province were used. To evaluate tumor growth, SW480 cells expressing CLCA4 were resuspended in a 1:1 mixture of phosphate-buffered saline and Matrigel matrix at various concentrations. The cell suspensions were injected subcutaneously into the left (control) and right (experimental) posterior flanks of the mice. Caliper measurements of tumor dimensions were taken periodically, and volumes were calculated using the formula: volume = (length × width^2^)/2. Upon reaching predefined ethical endpoints, the mice were euthanized. Tumor tissues were subsequently harvested for histopathological analyses.

To investigate the impact of CLCA4 overexpression on anti-PD-1 immunotherapy, CT-26 tumors were generated by injecting 2 × 10^6^ CLCA4-overexpressing tumor cells into the right flanks of Balb/c mice (6–8 weeks old). After 7 days, mice were intraperitoneally treated with either an *in vivo* blocking antibody against mouse PD-1 (Clone: 29F.1A2, BioXcell, Cat# BP0273) or a rat IgG2a isotype control antibody (Clone: 2A3, BioXcell, Cat# BP0089). Both antibodies were administered at a therapeutic dose of 150 μg per mouse 7, 10, 13, and 16 days after subcutaneous tumor cell injection. Tumor volume (L × W^2^/2) was measured every three days starting from day 7. On day 19, mice were sacrificed, and subcutaneous tumors were collected for analysis.

To establish a liver metastasis model, mice were initially anesthetized using 1.25% tribromoethanol solution (purchased from Nanjing Aibei Biotechnology Co., Ltd.; Cat# M2920) at a dosage of 200 μL for every 10 g of body weight. Subsequently, 6 × 10^5^ CT-26 cells, either control or those engineered to overexpress CLCA4, were introduced into the superior mesenteric vein during laparotomy, suspended in a 250 μL phosphate-buffered saline solution. Following the surgical procedure, the mice were given a 7-day recovery period. Beginning on day 7 post-surgery, they were then administered an *in vivo* blocking antibody against mouse PD-1 (Clone: 29F.1A2, sourced from BioXcell, Cat# BP0273) or a rat IgG2a isotype control antibody (Clone: 2A3, from BioXcell, Cat# BP0089) via intraperitoneal injection. The antibodies were given at a dosage of 150 μg per mouse on a schedule of 7, 10, 13, 16, and 19 days after the initiation of the liver metastasis model. On day 22, the mice were euthanized, and both body weight and liver weight measurements were taken. Subsequently, a section of the liver tissue, specifically from either the right anterior, right middle, or left anterior lobe, was extracted from each group for further analysis. The tissue samples were then fixed in 10% formalin, processed through a dehydration cycle, and embedded in paraffin for histological examination.

### Histological and immunohistological examinations

Immunohistochemistry was performed using standard protocols to further evaluate the relationship between CLCA4 and CSC biomarkers. Information on primary antibodies utilized in immunohistochemistry is provided in Table S6. Immunohistochemistry staining intensity was scored as “0” (negative), “1” (weak), “2” (moderate), or “3” (strong). The percentage of positive-stained cells was scored as “1” (≤10%), “2” (>10%–50%), “3” (>50%–75%), or “4” (>75%). An integrated immunohistochemistry score was obtained by multiplying the intensity and positive cell scores.

### Statistical analysis

Statistical analyses were conducted using SPSS 25.0 and GraphPad 9.0 software. Two-tailed Student's *t*-tests were utilized for comparisons between two independent groups. Two-way analysis of variance with mixed model testing was employed to evaluate *in vivo* tumorigenesis data. Quantitative data were presented as mean ± standard deviation. Statistically significant differences were defined as ∗*p* < 0.05 and ∗∗*p* < 0.01.

## Results

### CLCA4 expression was reduced in colorectal CSCs and drug-resistant cells

**Figure 1:**
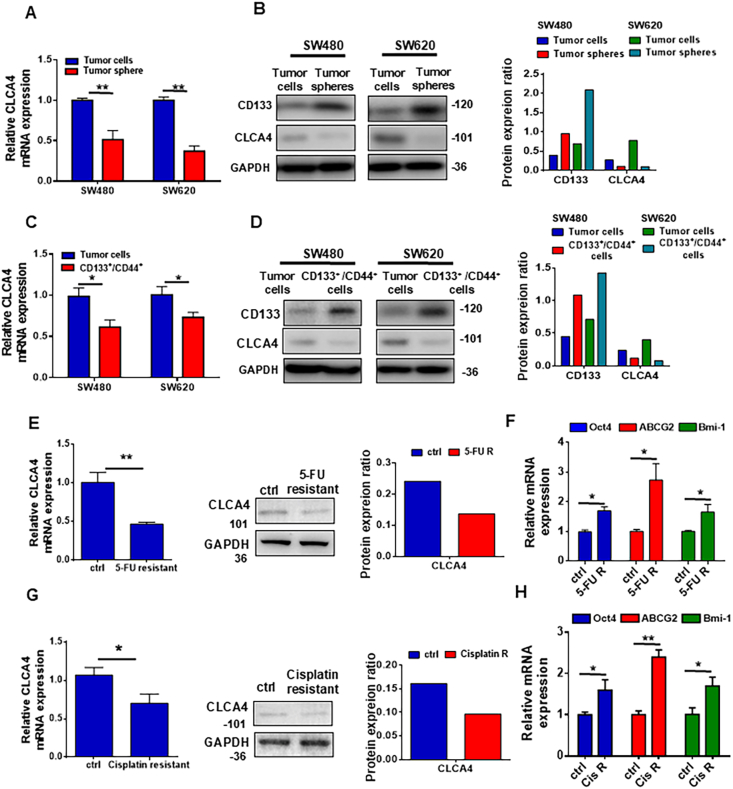
CLCA4 expression was reduced in colorectal cancer stem cells and drug-resistant cells. **(A**, **B**) The CLCA4 mRNA and protein expression levels in tumorspheres and adherent colorectal cancer (CRC) cells were compared by quantitative real-time PCR (qRT-PCR) and Western blotting. Right panels: Quantification of protein expression ratio. Unpaired two-tailed *t*-test (mean ± standard deviation). **(C**, **D**) The CLCA4 mRNA and protein expression levels in CD133^+^CD44^+^ CRC cells and adherent CRC cells were compared by qRT-PCR and Western blotting. Right panels: Quantification of protein expression ratio. Unpaired two-tailed *t*-test (mean ± standard deviation). **(E)** The expression of CLCA4 mRNA and protein in the 5-FU-resistant SW480 cells was evaluated using qRT-PCR and Western blotting. Right panels: Quantification of protein expression ratio. Unpaired two-tailed *t*-test (mean ± standard deviation). **(F)** The mRNA expression of stemness markers Oct4, ABCG2, and Bmi-1 in 5-FU-resistant SW480 cells was evaluated with qRT-PCR analysis. Unpaired two-tailed *t*-test (mean ± standard deviation). **(G)** The expression of CLCA4 mRNA and protein in the cisplatin-resistant SW480 cells was detected using qRT-PCR and Western blotting. Right panels: Quantification of protein expression ratio. Unpaired two-tailed *t*-test (mean ± standard deviation). **(H)** The mRNA expression of stemness markers Oct4, ABCG2, and Bmi-1 in cisplatin-resistant SW480 cells was evaluated with qRT-PCR analysis. Unpaired two-tailed *t*-test (mean ± standard deviation).

To determine the mechanisms underlying CLCA4 down-regulation in CRC pathogenesis, we first analyzed the expression of CLCA4 in CRC and adjacent non-cancerous tissues, and found that decreased CLCA4 expression was prevalent in cancer tissues. Reduced CLCA4 was correlated with larger tumors, metastasis, and poor survival outcomes, indicating its potential as a prognostic biomarker for aggressive CRC phenotypes (Fig. S1A–S1F; Tables S1 and S2). Given the close association between colorectal CSCs and chemoresistance, we decided to examine the expression levels of CLCA4 in colorectal CSCs. Our experiments revealed that CLCA4 expression was significantly down-regulated in CSC-enriched tumorspheres compared with traditional monolayer-cultured cells (Fig. 1A and B). When we compared CLCA4 expression in non-stem CRC cells with CRC cells expressing the well-established CSC markers CD133 and CD44, we found that CLCA4 was substantially decreased in the CRC cell populations (CD133^+^/CD44^+^) (Fig. 1C and D). To further investigate whether CLCA4 expression was also decreased in drug-resistant cell lines, we generated 5-FU- and cisplatin-resistant SW480 cells *in vitro* and found that CLCA4 expression was markedly reduced in both the 5-FU- and cisplatin-resistant cells, as measured by quantitative real-time PCR and Western blotting (Fig. 1E–G). Moreover, the mRNA levels of critical stemness-related genes (Bmi-1, ABCG2, and Oct4) were substantially higher in the 5-FU- and cisplatin-resistant cells compared with the control cells (Fig. 1F–H). Taken together, these results strongly imply that CLCA4 expression could be reduced in 5-FU- and cisplatin-resistant CRC cells exhibiting stem-like CSC characteristics.

### Ectopic expression of CLCA4 reduced the proliferation, invasion, and motility of CRC cells

**Figure 2:**
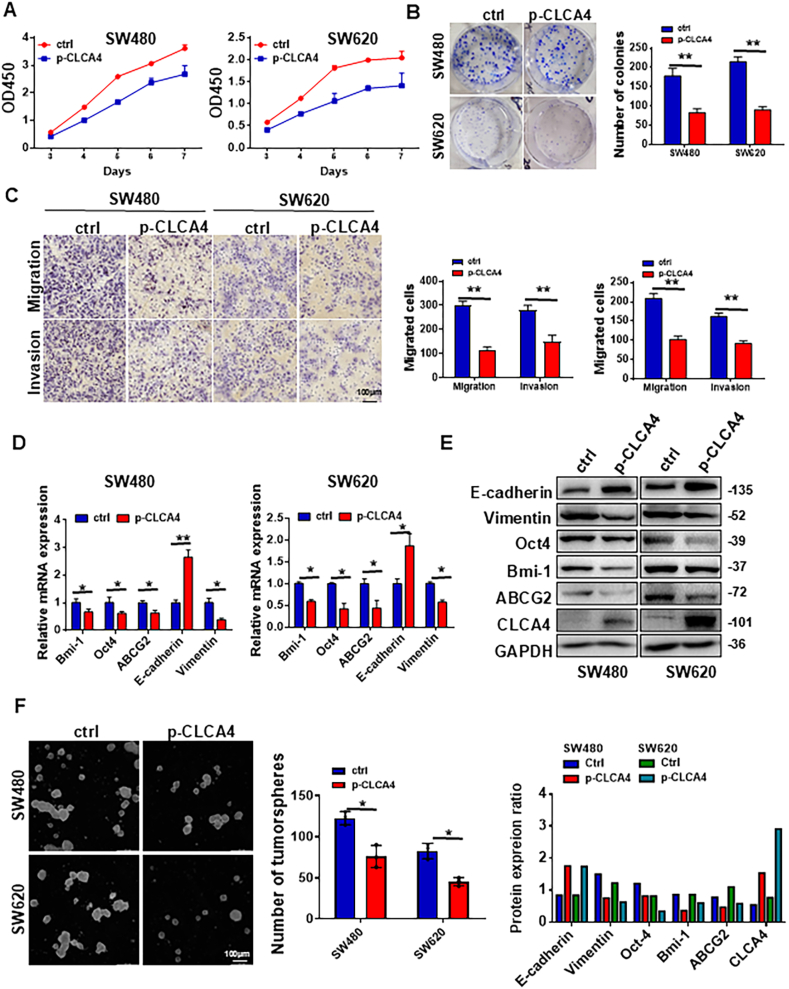
Ectopic expression of CLCA4 induced reduced proliferation, invasion, motility, and stem cell-like properties of colorectal cancer (CRC) cells. **(A)** CLCA4 reduced CRC cell proliferation. The survival rates were assessed by the CCK8 assay. **(B)** CLCA4 inhibited CRC cell proliferation. The colony sizes are shown in the left panels, and the colony numbers are shown in the right panels. Unpaired two-tailed *t*-test (mean ± standard deviation). **(C)** The motility and invasion of CLCA4 overexpressing CRC cells and control cells were detected by transwell migration and Boyden chamber invasion assays. Unpaired two-tailed *t*-test (mean ± standard deviation). **(D)** The mRNA expression of Bmi-1, Oct4, ABCG2, E-cadherin, and vimentin in CLCA4-expressing and control CRC cells was detected by PCR. Unpaired two-tailed *t*-test (mean ± standard deviation). **(E)** The protein expression of CLCA4, Bmi-1, Oct4, ABCG2, E-cadherin, and vimentin in CLCA4-expressing and control CRC cells was assessed using Western blotting analysis. Lower panels: Quantification of protein expression ratio. **(F)** CLCA4 suppressed CRC cell tumorsphere formation. The tumorsphere sizes are shown in the left panels, and the tumorsphere numbers are shown in the right panels. Unpaired two-tailed *t*-test (mean ± standard deviation).

To evaluate the effects of CLCA4 on proliferation, invasion, and motility, we established CRC cells with stable overexpression of CLCA4. CLCA4 overexpression was achieved in SW480 and SW620 CRC cells using lentiviral vectors (*p-CLCA4*, Ctrl). In terms of proliferation, CCK8 and colony formation assays showed significantly decreased viability and reduced colony formation in CLCA4-overexpressing CRC cells compared with controls (Fig. 2A and B). Transwell and Boyden chamber assays demonstrated that migration and invasion were significantly lower in CLCA4-overexpressing CRC cells than controls (Fig. 2C). Quantitative real-time PCR and Western blotting indicated that ectopic CLCA4 expression significantly increased the expression of epithelial marker E-cadherin and decreased the expression of mesenchymal marker vimentin in CRC cells (Fig. 2D and E). Together, these results suggest that CLCA4 overexpression could reduce CRC cell mobility and invasion *in vitro* by altering the expression of epithelial-mesenchymal transition-like cellular markers.

### Ectopic expression of CLCA4 reduced stem cell-like properties in CRC cells

To investigate the role of CLCA4 in regulating CSCs, we utilized the stably transfected CLCA4-overexpressing CRC cells and corresponding control cells. To determine the effect of altered CLCA4 expression on stemness genes in CRC cells, CSC markers were assessed by quantitative real-time PCR and Western blotting. Compared with controls, CLCA4-overexpressing cells showed substantially reduced expression of stemness genes, including Oct4, ABCG2, and Bmi-1, frequently used CSC markers (Fig. 2D and E). CSCs form spheroid tumorspheres under serum-free non-adhesive culture conditions and may be enriched under these conditions.^15^ Therefore, we examined the tumorsphere formation capacity of stably transfected CLCA4-overexpressing CRC cells using an *in vitro* tumorsphere formation assay. CLCA4-overexpressing cells formed fewer tumorspheres compared with vector control cells (Fig. 2F). These results indicate that CLCA4 could inhibit the self-renewal of colorectal CSCs and reduce their population size.

### CLCA4 decreased the number of tumor-initiating cells *in vivo*

**Figure 3:**
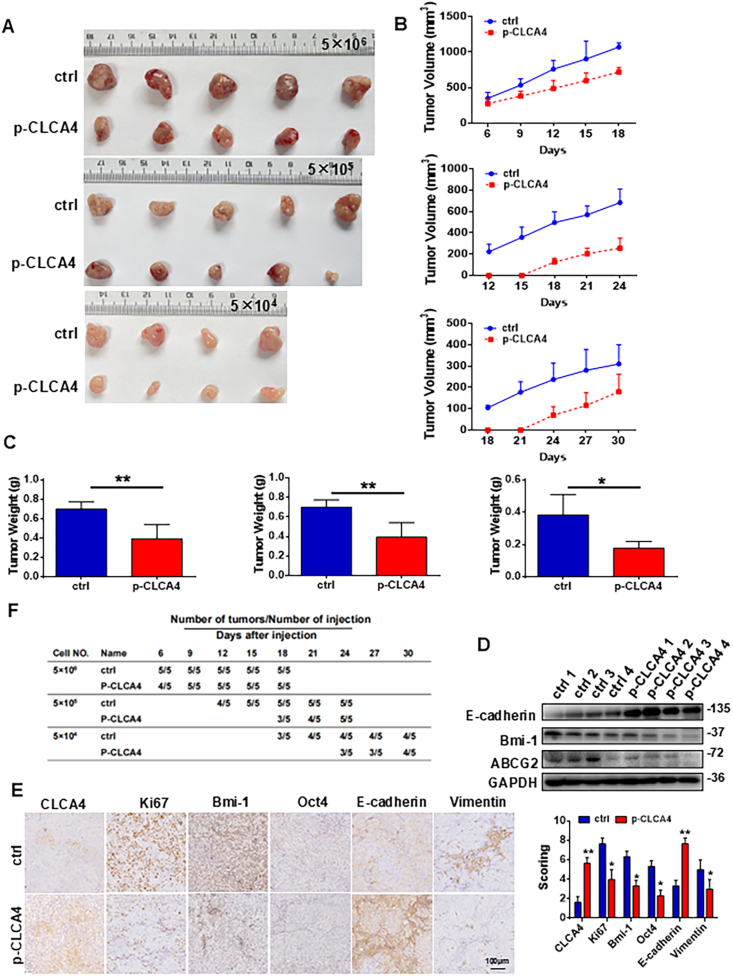
CLCA4 decreased the number of tumor-initiating cells *in vivo*. **(A)** An *in vivo* limiting dilution assay was performed by subcutaneous injection of CLCA4-overexpressing and vector control cells into nude mice, showing reduced tumor formation capacity of CLCA4-overexpressing cells. A representative image is shown. **(B)** Tumor growth curves are shown for SW480 cells stably expressing CLCA4 or empty vector control injected subcutaneously into nude mice (*n* = 5 per group) at doses of 5 × 10^6^, 5 × 10^5^, and 5 × 10^4^ cells. After tumors became palpable, CLCA4-expressing SW480 tumors displayed decreased growth rates compared with vector control tumors for all cell doses tested. Statistically significant differences in tumor growth were observed between CLCA4-overexpressing and vector control groups at each injected cell number. **(C)** Weights of tumors from nude mice injected with SW480 cells expressing CLCA4 or vector control. Unpaired two-tailed *t*-test (mean ± standard deviation). **(D)** The expression protein levels of ABCG2, Bmi-1, and E-cadherin in CLCA4-expressing or control tumors were determined through Western blotting analysis. **(E)** The expression protein levels of CLCA4, Ki67, Oct4, Bmi-1, E-cadherin, and vimentin in CLCA4-expressing or control tumors were evaluated through Western blotting analysis. Unpaired two-tailed *t*-test (mean ± standard deviation). **(F)** Tumors derived from CLCA4-expressing cells exhibited delayed onset of tumor formation and slower growth rates compared with control cells. Tumor development was monitored for 19–37 days following injection.

CLCA4 could suppress CRC stemness phenotypes. To further characterize the inhibitory effects of CLCA4 on CRC tumorigenicity, we performed limiting dilution assays in nude mice. Experiments injecting decreasing numbers of CLCA4-overexpressing versus control SW480 cells showed comparable tumor formation rates between the two groups. However, CLCA4 overexpression significantly suppressed tumor growth kinetics (Fig. 3A, B, F). With inoculation of 5 × 10^5^ or 5 × 10^4^ cells, the first palpable tumors appeared 6 days later in CLCA4-overexpressing cells than in control cohorts. After 19–37 days, CLCA4-overexpressing tumors were markedly smaller and lighter than controls (Fig. 3C). Immunohistochemistry or Western blotting analyses revealed decreased expression of CLCA4, Ki67, Oct4, and Bmi-1 in CLCA4-overexpressing xenografts compared with controls (Fig. 3D and E), indicating impaired proliferation and stemness. Taken together, our *in vitro* and *in vivo* data demonstrate that CLCA4 plays an important role in inhibiting the formation and propagation of tumor-initiating cells in CRC. The suppressive effects on tumor growth kinetics and stemness-associated markers further support the inhibitory function of CLCA4 in CRC.

### CLCA4 suppressed colorectal CSC expansion by down-regulating FAK-mediated signaling pathways

**Figure 4:**
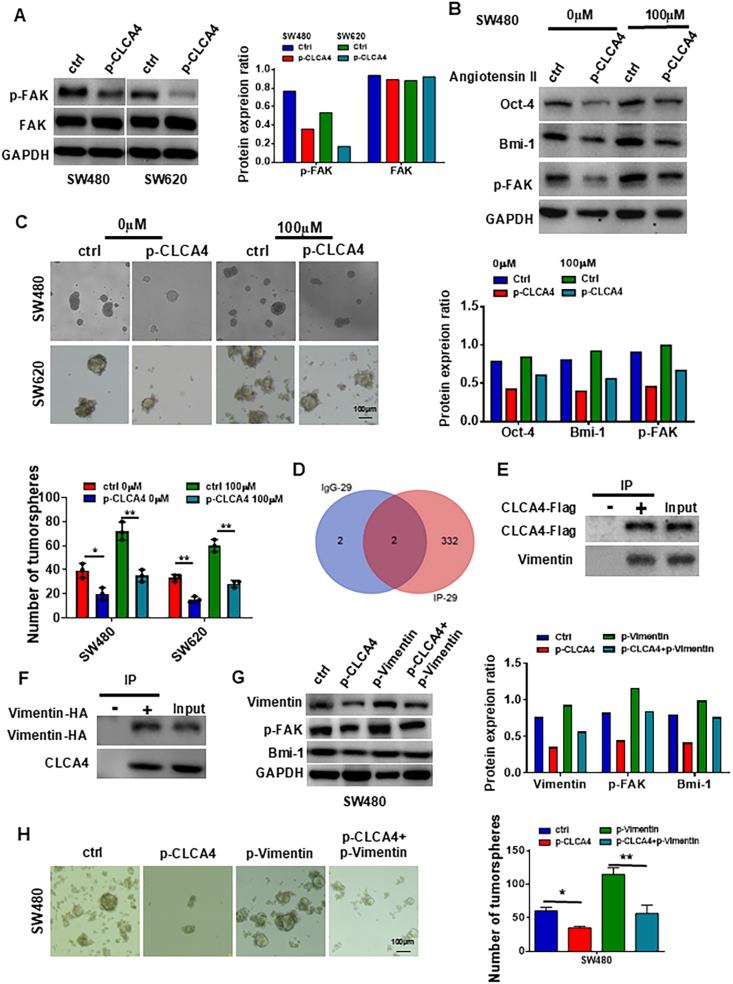
CLCA4 suppressed colorectal cancer stem cell expansion by interacting with vimentin to suppress FAK signaling pathways. **(A)** Western blotting analysis of FAK and p-FAK protein levels in control and CLCA4-overexpressing colorectal cancer (CRC) cells. Right panels: Quantification of protein expression ratio. **(B)** Western blotting analysis of stemness-related proteins and p-FAK in CLCA4-overexpressing cells treated with or without FAK agonist. Lower panels: Quantification of protein expression ratio. **(C)** Tumorsphere formation assay was performed to examine the tumorsphere formation ability in CLCA4-overexpressing cells treated with or without FAK agonist. One-way ANOVA with Tukey's multiple comparisons test (mean ± standard deviation). **(D)** Immunoprecipitation and IgG samples were analyzed by mass spectrometry. Proteins with unused >1.3 were filtered out, and keratin was removed. A total of 336 proteins were identified, including 334 proteins in immunoprecipitation samples and 4 proteins in IgG samples. **(E)** The immunoprecipitates of CLCA4 were purified using anti-Flag antibody and separated with SDS-PAGE, and the presence of vimentin was analyzed by Western blotting. Normal IgG was used as the negative control. **(F)** The immunoprecipitates of vimentin were purified using anti-HA antibody and separated with SDS-PAGE, and the presence of CLCA4 was analyzed by Western blotting. Normal IgG was used as the negative control. **(G)** The differences in protein levels (vimentin, Bmi-1, and p-FAK) among CRC cells transfected with different plasmids were analyzed by Western blotting. Right panels: Quantification of protein expression ratio. **(H)** Tumorsphere formation assay was performed to examine the tumorsphere formation ability among CRC cells transfected with different plasmids. One-way ANOVA with Tukey's multiple comparisons test (mean ± standard deviation).

To further explore the molecular mechanism by which CLCA4 inhibits CRC biological behaviors, we detected the downstream molecular signaling pathways affected by CLCA4 using RNA sequencing and Western blotting analyses, and found that overexpression of CLCA4 inhibited the FAK signaling pathway (Fig. 4A; Fig. S2A). After treatment with a FAK agonist, p-FAK expression was up-regulated, the expression of stemness-related proteins was partially restored (Fig. 4B), and the number of tumorspheres increased (Fig. 4C). Therefore, the FAK signaling pathway mediated the effect of decreased CLCA4 expression on the maintenance ability of CRC stem cells.

### Vimentin was critical for CLCA4 function in colorectal CSC expansion

To elucidate the mechanism by which CLCA4 maintains the stemness of colorectal CSCs, we employed mass spectrometry to identify potential proteins that directly interact with CLCA4. Comparative analysis of the immunoprecipitation sample and the IgG control sample revealed 336 differentially expressed proteins (Fig. 4D). Among these, we selected vimentin, which, in addition to its role in epithelial–mesenchymal transition, is also associated with CSC self-renewal, as a candidate CLCA4-interacting protein (Table S3). Co-immunoprecipitation experiments confirmed the reciprocal interaction between CLCA4 and vimentin (Fig. 4E and F). In addition, a GST pull-down assay demonstrated that CLCA4 interacted directly with vimentin *in vitro* (Fig. S5). To further understand how CLCA4 influenced vimentin, we treated cells with cycloheximide to inhibit protein production and monitored the effect of CLCA4 overexpression on vimentin stability. We found that in cells with increased CLCA4, vimentin levels decreased over time, notably at 12 h, compared with controls, suggesting that CLCA4 overexpression might enhance vimentin degradation (Fig. S6). To investigate whether vimentin mediated the CLCA4-induced inhibition of CRC stemness maintenance, we performed gain-of-function experiments and examined the role of vimentin in the stemness maintenance of CRC cells. Overexpression of vimentin increased the expression of stemness-related markers, promoted tumorsphere formation, and elevated p-FAK protein expression in CRC cells, which were opposite to the effects of CLCA4 overexpression (Fig. 4G). Furthermore, we found that ectopic expression of vimentin reversed the CLCA4-induced down-regulation of stemness-related markers, p-FAK expression, and inhibition of tumorsphere formation in CRC cells (Fig. 4G and H). We also utilized the TCGA CRC dataset, selecting two FAK-related pathways from the GeneCards database to analyze their correlation with vimentin expression. The results indicated a significant positive correlation between the FAK pathway and vimentin in CRC (Fig. S2B and S2C). Collectively, these findings suggest that CLCA4 could interact with vimentin and subsequently inactivate the FAK signaling pathway, thereby inhibiting the expansion of CSCs in CRC.

### CLCA4 expression was inversely correlated with stem cell-related marker expression in CRC

**Figure 5:**
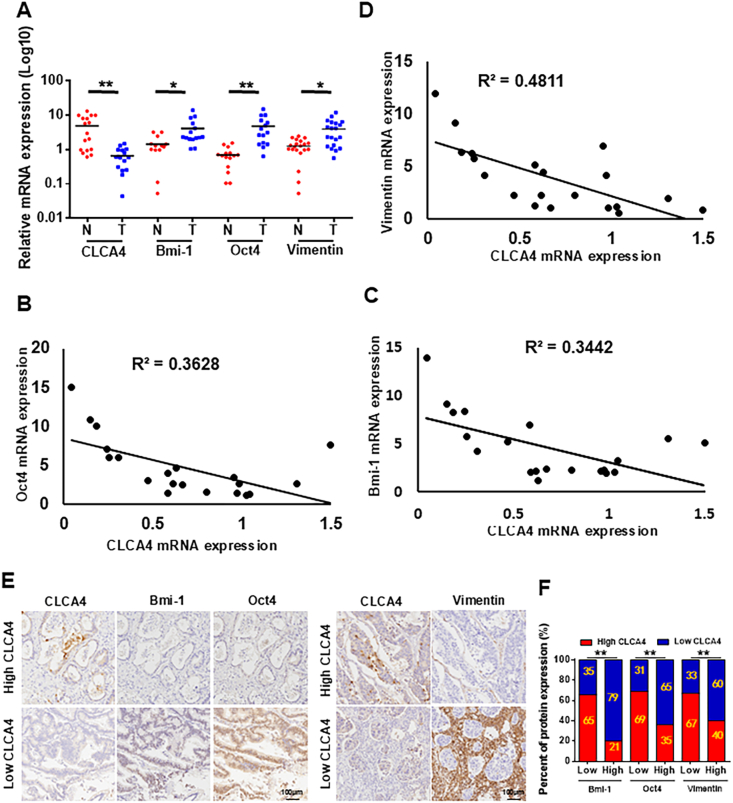
Correlation between CLCA4 expression and stem cell-related marker expression in colorectal cancer (CRC) patients. **(A)** The mRNA expression levels of CLCA4, Bmi-1, and Oct4 in CRC samples and control normal samples were detected by quantitative real-time PCR. N, normal; T, tumor. Unpaired two-tailed *t*-test (mean ± standard deviation). **(B**–**D)** An inverse correlation was observed between CLCA4 expression and Bmi-1, Oct4, and vimentin expression levels in CRC tissue samples. **(E, F)** CLCA4 expression was negatively correlated with the Bmi-1, Oct4, and vimentin expression levels in CRC tissue samples, as demonstrated by immunohistochemistry. *χ*^2^ test.

To investigate potential relationships between CLCA4 and stemness marker expression in CRC, we examined their mRNA levels in patient tissues. Total RNA was extracted from 20 adjacent normal and 20 CRC tissue samples, followed by quantitative real-time PCR analyses of the stem cell markers Bmi-1 and Oct4. Compared with normal samples, CRC tissues showed down-regulated expression of CLCA4 and up-regulated expression of the stemness markers (Fig. 5A). Furthermore, CLCA4 expression was negatively correlated with Bmi-1, Oct4, and vimentin levels (Fig. 5B–D). Consistent with mRNA data, immunohistochemistry of 100 CRC biopsies revealed that reduced CLCA4 protein expression was associated with elevated Bmi-1, Oct4, and vimentin (Fig. 5E and F; Table S4).

### CLCA4 overexpression enhanced the therapeutic effect of anti-PD-1

**Figure 6:**
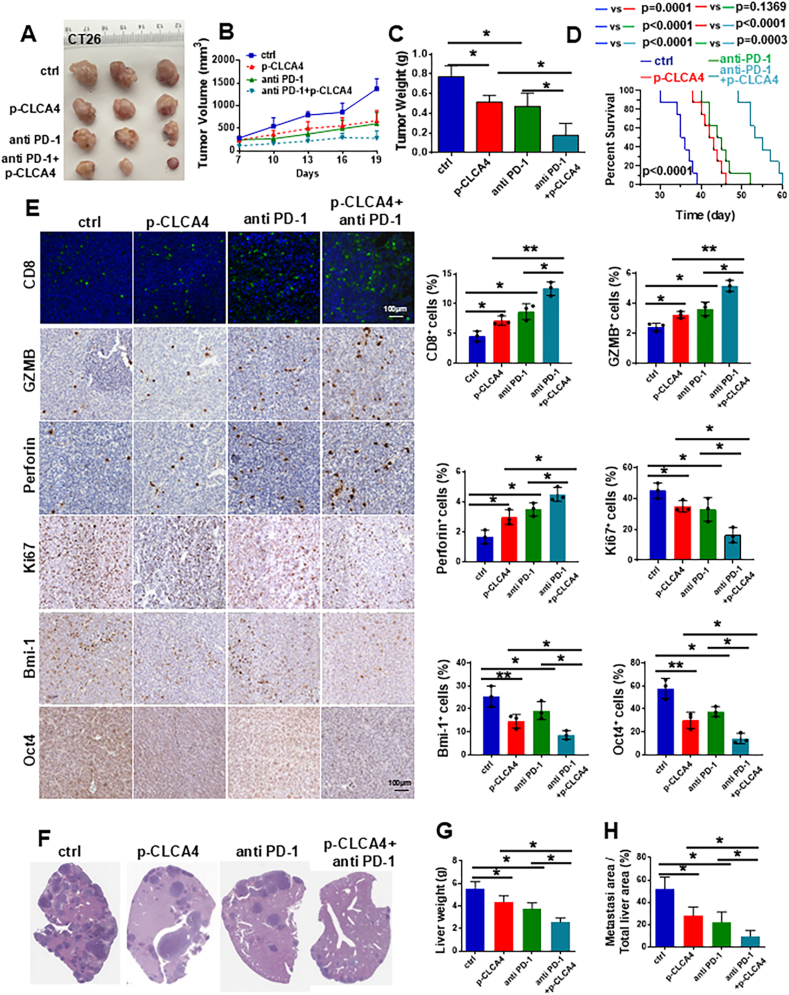
CLCA4 overexpression enhanced the therapeutic effect of anti-PD-1. **(A)***In vivo* tumorigenicity assay in nude mice was performed to detect the therapeutic effect of CLCA4 overexpression combined with anti-PD-1 antibody. **(B)** Tumor growth curve was monitored from nude mice with different treatment groups. **(C)** Weights of tumors from nude mice with different treatments were detected. One-way ANOVA with Tukey's multiple comparisons test (mean ± standard deviation). **(D)** Analysis of the survival times of mice in each group (*n* = 8 per group), and the experiment was terminated 60 days after tumor inoculation. Unpaired two-tailed *t*-test (mean ± standard deviation). **(E)** Immunofluorescence or immunohistochemistry staining was performed to detect the infiltration levels of CD8^+^ T cells, GZMB, Perforin^+^ cells, Ki67^+^, Bmi-1^+^, and Oct4^+^ cells in tumors from different treatment groups. One-way ANOVA with Tukey's multiple comparisons test (mean ± standard deviation). **(F)** Hematoxylin-eosin staining of liver metastases in each group. **(G)** Quantitative analysis of the liver weight in each group. One-way ANOVA with Tukey's multiple comparisons test (mean ± standard deviation). **(H)** Quantitative analysis of the liver metastasis area/total liver area in each group. One-way ANOVA with Tukey's multiple comparisons test (mean ± standard deviation).

Although CSCs play a significant role in the initiation and progression of tumors, their regulatory effects on the construction of the tumor immune microenvironment remain to be defined. We found that the decline in stemness transformation of CRC cells mediated by CLCA4 significantly reshaped the tumor microenvironment into an anti-tumor state. Notably, there was a significant infiltration of CD8^+^ cytotoxic T lymphocytes within the tumor (Fig. 6E), which strongly predicts a response to immune checkpoint blockade. This is consistent with the TIMER database prediction result that CD8^+^ T cell infiltration increases in tumor tissues with high CLCA4 expression (Fig. S3A). Further analysis of TCGA data showed that patients with high CLCA4 expression exhibited a better response to immune checkpoint inhibitors PD-1 and CTLA4 compared with the low CLCA4 expression group, and CLCA4 expression was positively correlated with immune activation-related pathways (BCR, TCR, chemokines, cytokines, TNF, *etc*.) and negatively correlated with the immune suppression-related TGF-β signaling pathway (Fig. S3B and S3C).

To validate the relationship between CLCA4 and immunotherapy, we performed *in vivo* animal experiments. The combination of p-CLCA4 and anti-PD-1 treatment significantly reduced tumor volume and weight, and slowed tumor growth compared with the control group and the single-agent treatment group (Fig. 6A–C). The survival analysis confirmed the superiority of the combined treatment (Fig. 6D). Consistently, within the CT-26 model of liver metastasis, the synergistic effect of the combined therapy led to a more pronounced suppression of hepatic metastases than that of monotherapy (Fig. 6F). There was a significant reduction in both the liver weight and the ratio of the metastatic area to the entire liver area in the group receiving the combined treatment (Fig. 6G and H). To further evaluate the synergistic therapeutic effects of the combined treatment, immunofluorescence staining and immunohistochemistry staining revealed a significant increase in CD8^+^ T cell infiltration in the combination treatment group, and there was a significant rise in the proportion of CD8^+^ T cells secreting granzyme GZMB and perforin, the proliferation of tumor cells, as indicated by Ki67, was reduced, and the stemness transformation in tumor tissue following the combined treatment was significantly diminished (Fig. 6E). Inspired by the TCGA data, we further paid attention to chemokines and detected the expression chemokine CXCL10, and observed a higher expression of CXCL10 in tumors versus the control (Fig. S7). In CRC, numerous studies have reported that up-regulated expression of chemokine CXCL10 is correlated well with the increased cytotoxic T lymphocyte infiltration^16^, ^17^, ^18^ and enhanced chemo- and anti-PD-1-based therapy.^19^^,^^20^ These data suggest that CLCA4-mediated suppression of stemness and increased CXCL10 expression could lead to a decrease in tumor malignancy and promote the efficacy of anti-PD-1 treatment in CRC.

## Discussion

Our study provides compelling evidence for the tumor-suppressive role of CLCA4 in CRC and its potential implications in clinical management and treatment. We demonstrated that diminished CLCA4 expression was a prevalent occurrence in CRC tissues and was associated with aggressive phenotypes, including advanced tumor stage, invasion, and metastasis. Importantly, our data indicate a significant correlation between reduced CLCA4 expression and unfavorable prognostic outcomes, emphasizing the potential utility of CLCA4 as a prognostic marker in CRC. These findings further link CLCA4 down-regulation to poor prognosis in various cancers, underscoring the clinical significance of CLCA4 as a biomarker for cancer progression and patient outcomes.^21^, ^22^, ^23^

Our investigation into the mechanistic underpinnings of CLCA4 in CRC elucidates its inhibitory effects on CSC properties and tumor-initiating capability. Notably, CLCA4 overexpression led to decreased stemness marker expression, inhibited tumorsphere formation, and suppressed tumorigenicity *in vivo*, implicating CLCA4 as a potential regulator of CRC stemness and tumorigenic potential. These findings are in line with prior research identifying the critical role of CSCs in driving tumorigenesis, metastasis, and therapeutic resistance in CRC.^24^, ^25^, ^26^, ^27^, ^28^

Furthermore, our research identifies CLCA4 as a key regulator of colorectal CSCs, acting through the suppression of the FAK signaling pathway, which is vital for cancer cell survival and proliferation. The negative impact of CLCA4 overexpression on the FAK pathway is pivotal in reducing CSC characteristics, as indicated by the reversal of these effects with a FAK agonist. These findings are in accordance with existing literature linking FAK signaling to CSC maintenance and tumorigenicity in various cancer types, emphasizing the significance of the FAK pathway in driving cancer stemness and progression.^29^, ^30^, ^31^, ^32^, ^33^ Vimentin emerges as an essential intermediate in CLCA4 regulatory function, with our co-immunoprecipitation studies confirming their interaction and suggesting its influence on CSC self-renewal. The TCGA data correlation between FAK signaling and vimentin expression supports our experimental findings, pointing to a cooperative role in CSC biology. In essence, our study proposes that CLCA4, through its interaction with vimentin, deactivates the FAK pathway, presenting a potential therapeutic approach for CRC. The strategic targeting of the CLCA4-vimentin-FAK axis holds promise for combating CSC-driven tumorigenesis and chemoresistance, warranting further investigation for clinical application.

Moreover, CSCs are known to initiate and maintain specific immune cell clusters within the tumor microenvironment, including tumor-associated macrophages and regulatory T cells, which contribute to a state of immunosuppression that aids in immune evasion.^34^, ^35^, ^36^ The precise regulatory mechanisms of CSC-immune system interactions remain to be fully elucidated. Here, we propose that effectively targeting CSCs has the potential to reconfigure their surrounding microenvironment, which could subsequently boost the effectiveness of immunotherapeutic strategies. In this study, we conducted experiments using a microsatellite-stable CRC mouse model to assess the synergistic impact of CLCA4 modulation and anti-PD-1 immunotherapy. The strategic reduction of stemness properties through CLCA4 targeting significantly improved the response to anti-PD-1 treatment. We also discovered that CLCA4 may play a previously unrecognized role in promoting the infiltration of tumor-infiltrating lymphocytes. CLCA4 boosts CD8^+^ T cell infiltration by secreting the chemokine CXCL10. It also shifts the tumor microenvironment from immunosuppressive to immunostimulatory by up-regulating immune-activating pathways, such as BCR, TCR, cytokines, and TNF, and down-regulating TGF-β signaling. These changes enhance the therapeutic effect of immunotherapy. Collectively, these findings offer promising insights, suggesting that interventions focused on CLCA4 could increase the susceptibility of microsatellite-stable CRC to immunotherapy.

In summary, our study explored the tumor-suppressive role of CLCA4 in CRC and its potential as a therapeutic target. CLCA4 down-regulation renders CRC cells aggressive phenotypes and chemoresistance, while its up-regulation inhibits the stem-like properties of CRC cells. CLCA4 interacts with vimentin and regulates FAK signaling and CSC maintenance. Increased CLCA4 expression enhances the therapeutic effect of anti-PD-1. Therefore, modulation of CRC stemness could reshape the tumor immune microenvironment and improve immunotherapy, thus providing a foundation for future research into the clinical application of CLCA4 in CRC management.

## CRediT authorship contribution statement

**Fang Wei:** Writing – review & editing, Writing – original draft, Supervision, Resources, Project administration, Methodology, Investigation, Funding acquisition, Formal analysis, Data curation, Conceptualization. **Qi Zou:** Methodology, Investigation, Formal analysis, Data curation. **Qihui Sun:** Formal analysis, Data curation. **Tingting Jiang:** Formal analysis, Data curation. **Tian Cai:** Formal analysis, Data curation. **Xiaojia Li:** Resources, Project administration, Methodology, Investigation, Formal analysis, Data curation. **Keping Xie:** Writing – review & editing, Writing – original draft, Supervision, Resources, Project administration, Formal analysis, Conceptualization.

## Funding

The work is partly supported by the 10.13039/501100001809National Natural Science Foundation of China (No. 81972780) and the 10.13039/501100003453Natural Science Foundation of Guangdong Province, China (No. 2020A1515010051).

## Conflict of interests

All authors declared no conflict of interests. Keping Xie is the associate editor of *Genes & Diseases*, but he has no involvement in the peer-review of this article and has no access to information regarding its peer-review.
