# Supplementary material for: Chloride channel accessory 4 suppresses stem cell-like properties of colorectal cancer and enhances anti-PD-1 immunotherapy
Source: Genes Dis. 2025 Sep 20;13(3):101859. doi: 10.1016/j.gendis.2025.101859 (PMC12886529; doi:10.1016/j.gendis.2025.101859)
Supplement: Multimedia component 1 [file mmc1.pdf]

Chloride channel accessory 4 suppresses stem cell-like properties of colorectal cancer and enhances anti-PD-1 immunotherapy

Fang Wei[a,b,c,d], Qi Zou[a,b,c,d], Qihui Sun[a,b,d], Tingting Jiang[a,b], Tian Cai[a,b], Xiaojia Li[a,b], Keping Xie[a,b,c,*], mcxiekeping@scut.edu.cn

[a] Center for Pancreatic Cancer Research, The South China University of Technology School of Medicine, Guangzhou, Guangdong 510006, China

[b] Department of Immunology and Pathology, The South China University of Technology School of Medicine, Guangzhou, Guangdong 510006, China

[c] Guangzhou First People's Hospital and The Second Affiliated Hospital, South China University of Technology School of Medicine, Guangzhou, Guangdong 510180, China

[*] Corresponding author. Center for Pancreatic Cancer Research and Department of Immunology, South China University of Technology School of Medicine, 382 Waihuan Road, Guangzhou, Guangdong 510006, China.

[d] These authors contributed equally to this work.

Supplementary Materials

1. **Supplementary Figures**
2. **Supplementary Tables**

1. Supplementary Figures

Low CLCA4 expression commonly observed in CRC tissues and correlated with aggressive and unfavorable prognostic phenotypes of CRC

IHC was utilized to evaluate CLCA4 protein expression in 110 formalin-fixed paraffin-embedded CRC specimens and 20 non-cancerous colorectal epithelial specimens. Low CLCA4 expression was detected in 98 of 110 (89%) CRC samples and 18 of 20 (90%) non-cancerous samples, respectively (**Fig. S1A–S1B; Table S1**). Quantitative real-time PCR (qRT-PCR) revealed significantly decreased CLCA4 expression in CRC tissues *vs* matched adjacent normal tissues (**Fig. S1C**). CLCA4 downregulation occurred frequently in CRC compared to non-cancerous colorectal epithelium. Furthermore, IHC demonstrated CLCA4 localization in both the membrane and cytoplasm of cancer cells (**Fig. S1A and S1D**). Associations between CLCA4 expression and clinicopathologic features were analyzed in 110 CRC cases (**Table S2**). No significant correlations were observed between CLCA4 levels and patient age, gender, or lymph node invasion status (N classification). However, CLCA4 expression significantly correlated with tumor size (T classification), metastasis (M classification), and overall stage. Specifically, reduced CLCA4 expression occurred frequently in T4, M1 and stage III-IV tumors as compared to T2-T3, M0 and stage I-II tumors, indicating that low CLCA4 associated with aggressive CRC phenotypes and contributed to CRC invasion and metastasis (**Fig. S1D–S1E; Table S2**). Given links between low CLCA4 expression and advanced CRC, we further assessed the prognostic value of CLCA4 expression. Kaplan-Meier analysis demonstrated a positive correlation between CLCA4 levels and overall survival. Patients with low CLCA4 expression had significantly poorer overall survival than those with high expression (**Fig. S1F**).

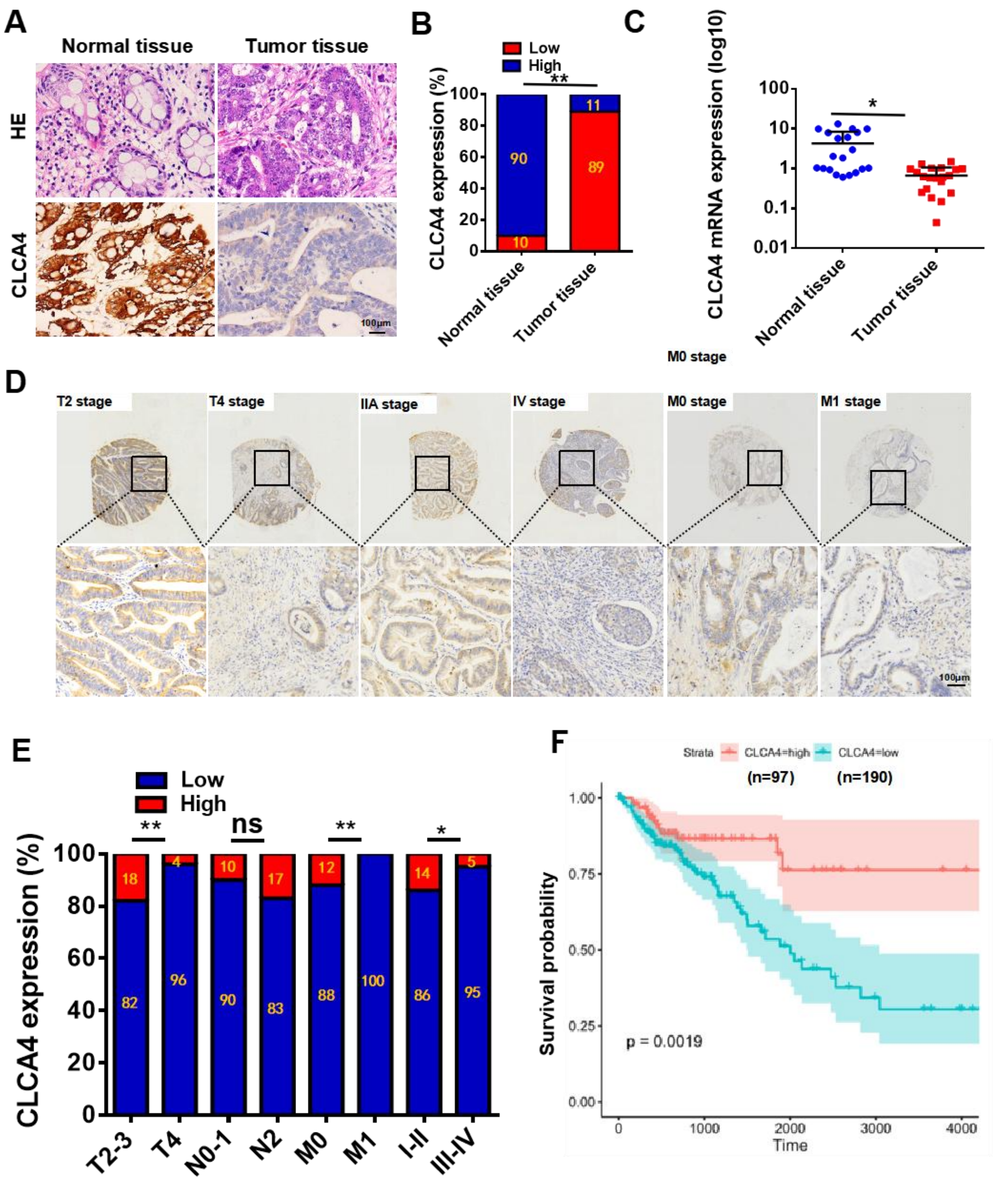

Figure S1 Low CLCA4 expression commonly observed in colorectal cancer tissues. **(A)** IHC analysis of CLCA4 protein levels in CRC and adjacent normal tissues. **(B)** CLCA4 expression was significantly lower in CRC than that in noncancerous samples. **(C)** qRT-PCR analysis: CLCA4 expression was significantly lower in the CRC biopsies than that in the noncancerous biopsies. **(D)** Representative images of CLCA4 expression in CRC biopsies of different TNM stages. **(E)** Quantitative analysis of differential CLCA4 expression patterns stratified by clinicopathological characteristics.

(F) Kaplan-Meier analysis of overall survival in a cohort of 287 colorectal cancer patients stratified by CLCA4 expression levels (high or low) using The Cancer Genome Atlas database.

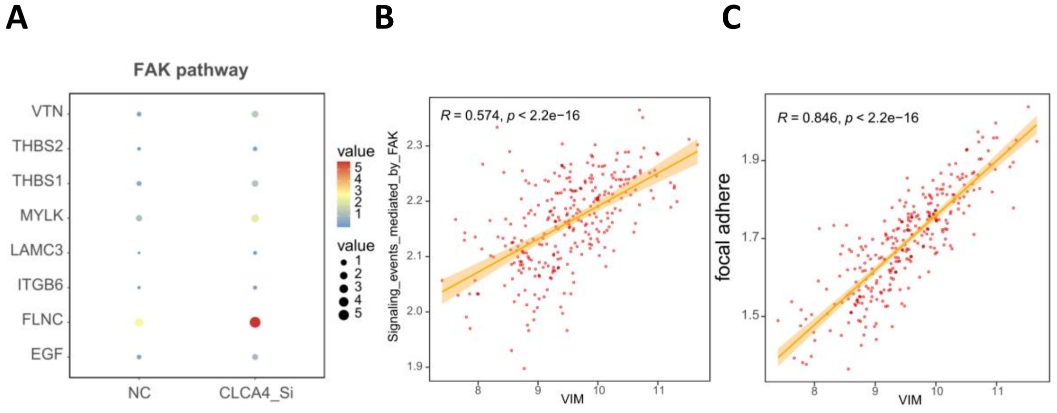

Figure S2 Analysis of the Correlation between the FAK Pathway and vimentin. **(A)** RNA-seq analysis of the elevated expression of genes associated with the FAK pathway in the control and siCLCA4 group. **(B–C)** TCGA colorectal cancer dataset analysis of the positive correlation between the selected two FAK-related pathways from the GeneCards database with the expression of vimentin (“VIM”) gene in colorectal cancer.

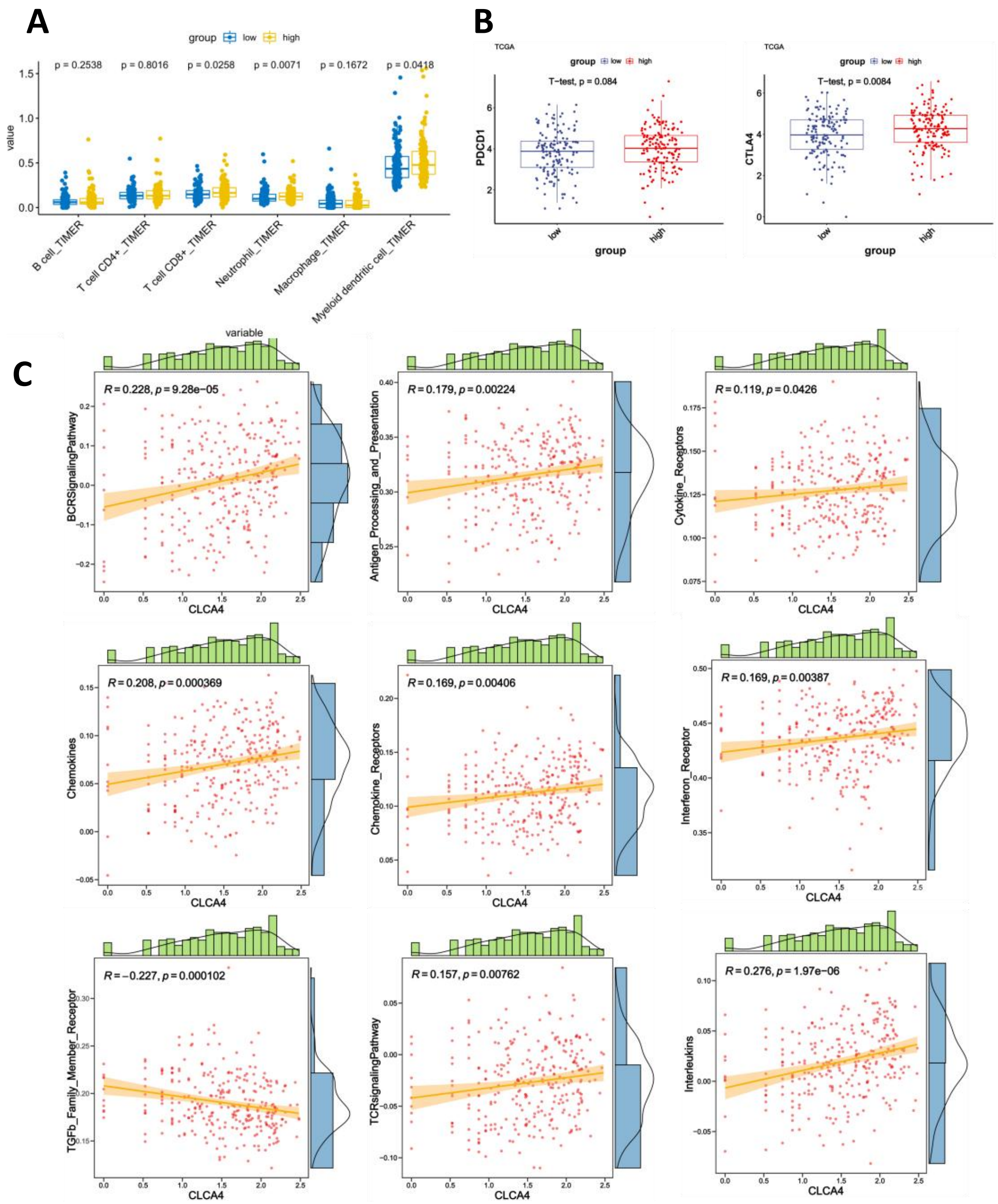

Figure S3 The Relationship between CLCA4 Expression and Immune response-related parameters. **(A)** TIMER database analysis of the effect of CLCA4 expression on immune cell infiltration. **(B)** TCGA database analysis of CLCA4 expression and response to immune checkpoint inhibitor therapy. **(C)** TCGA database analysis of the enrichment of immune response-related pathways.

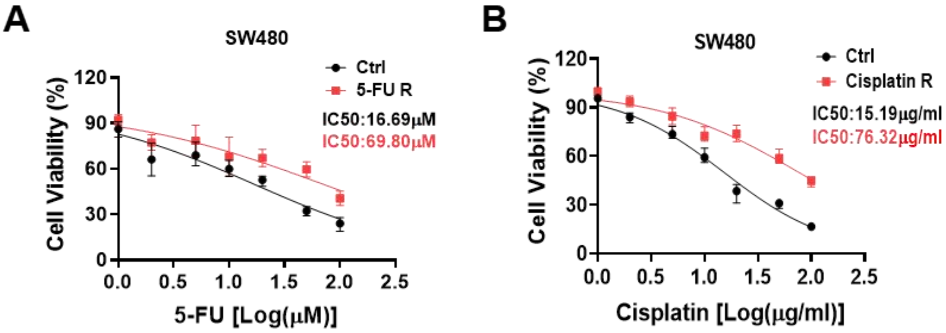

Figure S4 Cell viability assays showing resistance of SW480 cells to 5-FU and cisplatin. **(A)** Left panel displayed the dose-response curves for 5-FU in control ("Ctrl") and 5-FU-resistant ("5-FU R") SW480 cells. The IC50 values are indicated for both cell types. **(B)** Right panel showed the dose-response curves for cisplatin in control ("Ctrl") and cisplatin-resistant ("Cisplatin R") SW480 cells, with corresponding IC50 values provided.

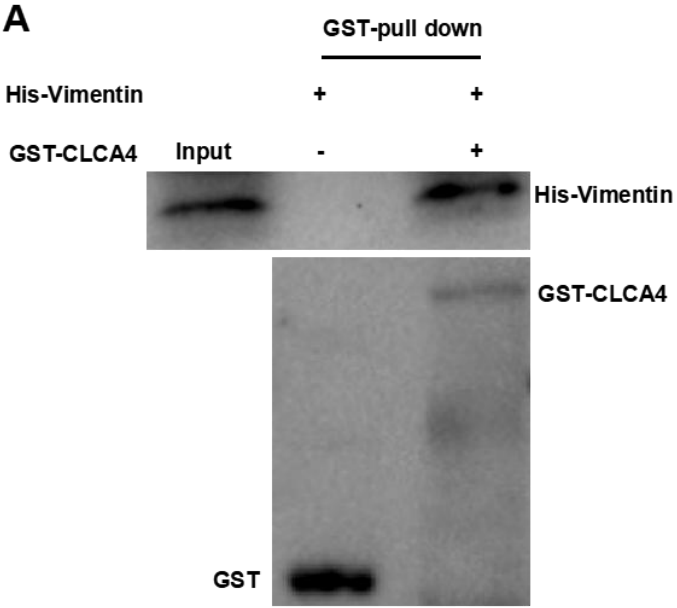

Figure S5 CLCA4 interacted with Vimentin. **(A)** CLCA4 binding to Vimentin using the GST pull-down assay. The control GST and GST-CLCA4 fusion protein were purified with GST-agarose, after being mixed with purified His-Vimentin protein and the interaction was detected by Western blotting.

A

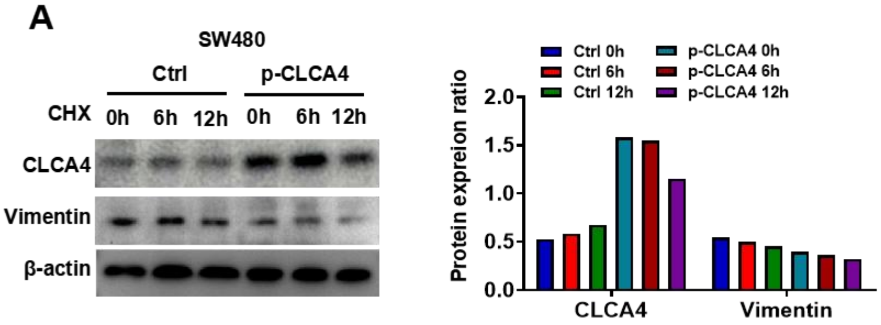

Figure S6 Impact of CLCA4 Overexpression on Vimentin Stability in SW480 Cells. **(A)** Western blot analysis of CLCA4 and Vimentin protein levels in SW480 cells with or without CLCA4 overexpression, treated with cycloheximide (“CHX”) to inhibit protein synthesis. Right panels: Quantification of protein expression ratio.

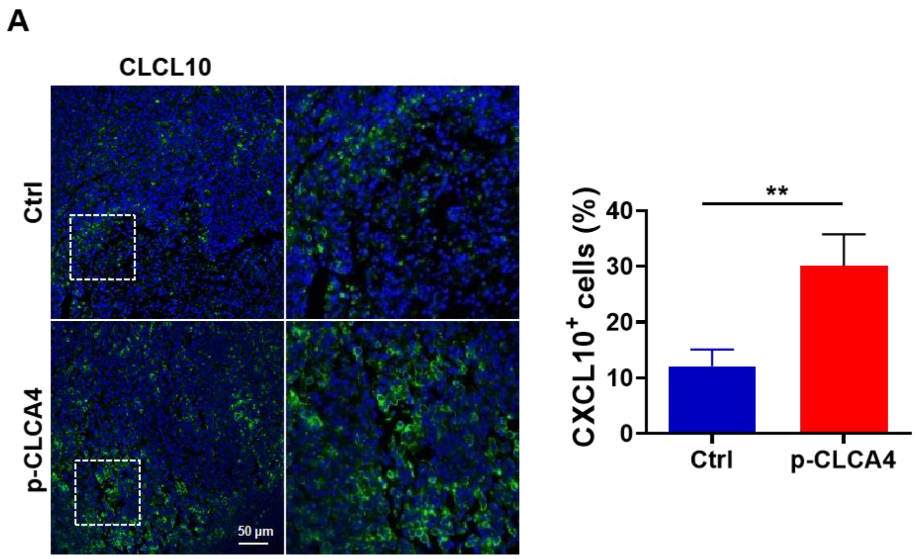

Figure S7 Increased CXCL10 expression in CLCA4-overexpressing CT26 tumors. **(A)** CXCL10 expression in tumor tissues was measured by immunofluorescence.

2. Supplementary Tables

Table S1 CLCA4 expression in 110 colorectal cancer (CRC) specimens and 20 non-cancerous colorectal epithelial specimens.

Variables	*n*	CLCA4 expression		X^2	***P***
		Low （*n*,%）	High （*n*,%）		
Non-cancerous tissue	20	（2,10）	（18,90）	124.8	<0.0001
CRC	110	（98,89）	（12,11）		

Table S2 Associations between CLCA4 expression and clinicopathologic features were analyzed in 110 CRC cases.

**Variables**	***n***	**CLCA4 expression**		***X*²**	***P***
		**Low （*n*,%）**	**High （*n*,%）**		
Sex					
Female	36	（33,92）	（3,8）	0.8889	0.3458
Male	74	（65,88）	（9,12）		
Age					
<50	33	（30,91）	（3,9）	0.4789	0.4889
≥50	77	（68,88）	（9,12）		
T classification					
T2-3	57	（47,82）	（10,18）	10.01	0.0016
T4	53	（51,96）	（2,4）		
N classification					
N0-1	98	（88,90）	（10,10）	2.098	0.1475
N2	12	（10,83）	（2,17）		
M classification					
M0	102	（90,88）	（12,12）	12.77	0.0004
M1	8	（8,100）	（0,0）		
Clinical stage					
I-II	71	（61,86）	（10,14）	4.711	0.03
III-IV	39	（37,95）	（2,5）		

Table S3 TOP 20 differentially expressed proteins were identified by mass spectrometry between IP sample and IgG sample.

Accession	Gene	Name	Unused IP	Unused IgG	Peptide IP	Peptide IgG	Spectra IP	Spectra IgG
P35579	MYH9	Myosin-9	132.83	NA	62	NA	151	NA
P35580	MYH10	Myosin-10	93.73	NA	59	NA	138	NA
Q13813	SPTN1	Spectrin alpha chain, non-erythrocytic 1	98.78	NA	47	NA	96	NA
P08670	**Vim**	Vimentin	65.47	NA	33	NA	129	NA
P63261	ACTG	Actin, cytoplasmic 2	51.23	NA	27	NA	118	NA
P60709	ACTB	Actin, cytoplasmic 1	2	NA	26	NA	125	NA
Q01082	SPTB2	Spectrin beta chain, non-erythrocytic 1	44.49	NA	20	NA	30	NA
Q7Z406	MYH14	Myosin-14	10.92	NA	17	NA	21	NA
P07437	TBB5	Tubulin beta chain	32.08	NA	16	NA	98	NA
P68371	TBB4B	Tubulin beta-4B chain	4.1	NA	15	NA	94	NA
P13639	EF2	Elongation factor 2	33.56	NA	14	NA	33	NA
P38646	GRP75	Stress-70 protein, mitochondrial	25.44	NA	13	NA	37	NA
P18124	RL7	60S ribosomal protein L7	20.27	NA	12	NA	20	NA

P36578	RL4	60S ribosomal protein L4	21.56	NA	12	NA	25	NA
P25705	ATPA	ATP synthase subunit alpha, mitochondrial	22.65	NA	12	NA	21	NA
P08238	HS90B	Heat shock protein HSP 90-beta	25.25	NA	12	NA	28	NA
Q9NR30	DDX21	Nucleolar RNA helicase 2	21.93	NA	12	NA	19	NA
P63244	RACK1	Receptor of activated protein C kinase 1	23.01	NA	11	NA	28	NA
P17844	DDX5	Probable ATP-dependent RNA helicase DDX5	21.22	NA	10	NA	33	NA
P07900	HS90A	Heat shock protein HSP 90-alpha	4.5	NA	9	NA	17	NA

“Unused”, protein score; “NA”, undetected

Table S4 Association of CLCA4 expression and stem cell marker in 100 CRC tissues.

Variables	n	CLCA4 expression			
		**High (*n*, %)**	**Low (*n*, %)**	χ^2	***P***
Bmi-1					
Low expression	62	40 (64.5)	22 (35.5)	4.223	<0.0001
High expression	38	8 (21)	30 (79)		
Oct4					
Low expression	52	36 (69.2)	16 (30.8)	3.385	0.0007
High expression	48	17 (35.4)	31 (64.6)		
Vimentin					
Low expression	55	37 (67.3)	18 (32.7)	2.727	0.0064
High expression	45	18 (40)	27 (60)		

Table S5. Primers for qRT-PCR analysis

Gene	Forward primer (5’ -> 3’)	Reverse primer (5’ -> 3’)
CLCA4	CCTGCTGCACCAGTCAAATAC	ACGTAGAAGCTGTAGTCACCATA
Bmi-1	CGTGTATTGTTCGTTACCTGGA	TTCAGTAGTGGTCTGGTCTTGT
ABCG2	CAGGTGGAGGCAAATCTTCGT	ACCCTGTTAATCCGTTCGTTTT
Oct4	CTTGCTGCAGAAGTGGGTGGAGGAA	CTGCAGTGTGGGTTTCGGGCA
E-cadherin	TGCCCAGAAAATGAAAAAGG	GTGTATGTGGCAATGCGTTC
Vimentin	GAGAACTTTGCCGTTGAAGC	GCTTCCTGTAGGTGGCAATC
GAPDH	ACCCAGAAGACTGTGGATGG	TCTAGACGGCAGGTCAGGTC

Table S6 List of antibodies and suppliers used for WB and IHC.

Antibody	Isotype	Suppliers	Cat. No
CLCA4	Rabbit IgG	EMD Millipore	MABN1581
Ki67	Rabbit IgG	Abcam	AB16667
ABCG2	Rabbit IgG	Bioworld	LM0796
Bmi-1	Rabbit IgG	Bioworld	MB9014
Oct4	Rabbit IgG	Bioworld	BS70993
E-cadherin	Rabbit IgG	CST	3195
Vimentin	Rabbit IgG	CST	5741S
FAK	Rabbit IgG	CST	71433S
p-FAK	Rabbit IgG	Invitrogen	700255
CD8	Rabbit IgG	CST	98941S
GZMB	Rabbit IgG	CST	44153S
Perforin	Rabbit IgG	CST	31647S
GAPDH	Mouse IgG	Proteintech	60004-1-Ig
CXCL10	Rabbit IgG	Proteintech	10937-1-AP
